# A case report of atypical ethylmalonic encephalopathy with peripheral neuropathy

**DOI:** 10.1111/cns.14072

**Published:** 2023-01-04

**Authors:** Guan‐Qing Wang, Ze‐Yu Zhao, Yan‐Bin Li

**Affiliations:** ^1^ Department of Neurology, Shandong Provincial Qianfoshan Hospital, School of Clinical Medicine Weifang Medical University Weifang China; ^2^ Department of Neurology The First Affiliated Hospital of Shandong First Medical University Jinan China; ^3^ Shandong Institute of Neuroimmunology Jinan China

Dear Editor,

Ethylmalonic encephalopathy (EE; OMIM #602473) is an autosomal recessive disorder caused by mutations in ethylmalonic encephalopathy protein 1 (ETHE1)^1^ and is characterized by global developmental delay, infantile hypotonia, seizures, and microvascular damage.^2^ Here, we are the first to report a case of EE with peripheral neuropathy in an adolescent male patient with a novel compound heterozygous c.475C>T (p.R159C) variant of ETHE1.

A 12‐year‐old boy was referred to our hospital with progressive gait impairment and growth retardation from 8 years of age (Video S1). He was the first child of healthy non‐consanguineous Chinese parents with an unremarkable family history. The patient was born at term following an uneventful pregnancy and delivery. On admission, the patient was 137 cm tall and weighed 28.5 kg. The patient did not show any behavioral changes, cognitive symptoms, or incontinence. Physical examination showed active tendon reflexes in both lower extremities, positive bilateral Babinski and Chaddock signs, weakness of dorsiflexion of the right foot, and power of 5−/5 in both lower limbs and 5/5 in the other two limbs. No sensory or cranial nerve abnormalities were noted. Metabolic tests showed elevated levels of ethylmalonic acid (EMA) [10.29; reference range (r.v), 0.00–6.20], glycolic acid (10.84; r.v, 0.00–2.20), isovaleryl glycine (7.01; r.v, 0.00–0.40), palmitic acid (405.69; r.v, 0.00–13.80), and phosphoric acid (273.87; r.v, 0.00–43.00) in urine using gas chromatography–mass spectrometry. No significant abnormalities were found in blood metabolic analysis using high‐performance liquid chromatography–tandem mass spectrometry. Neuroimaging revealed hyperintensity in the bilateral putamen on brain MRI, but normal hyperintensity on spinal MRI (Figure 1A). Nerve conduction studies revealed that the bilateral peroneal nerve motor responses showed reduced amplitudes (Table 1).

**FIGURE 1:**
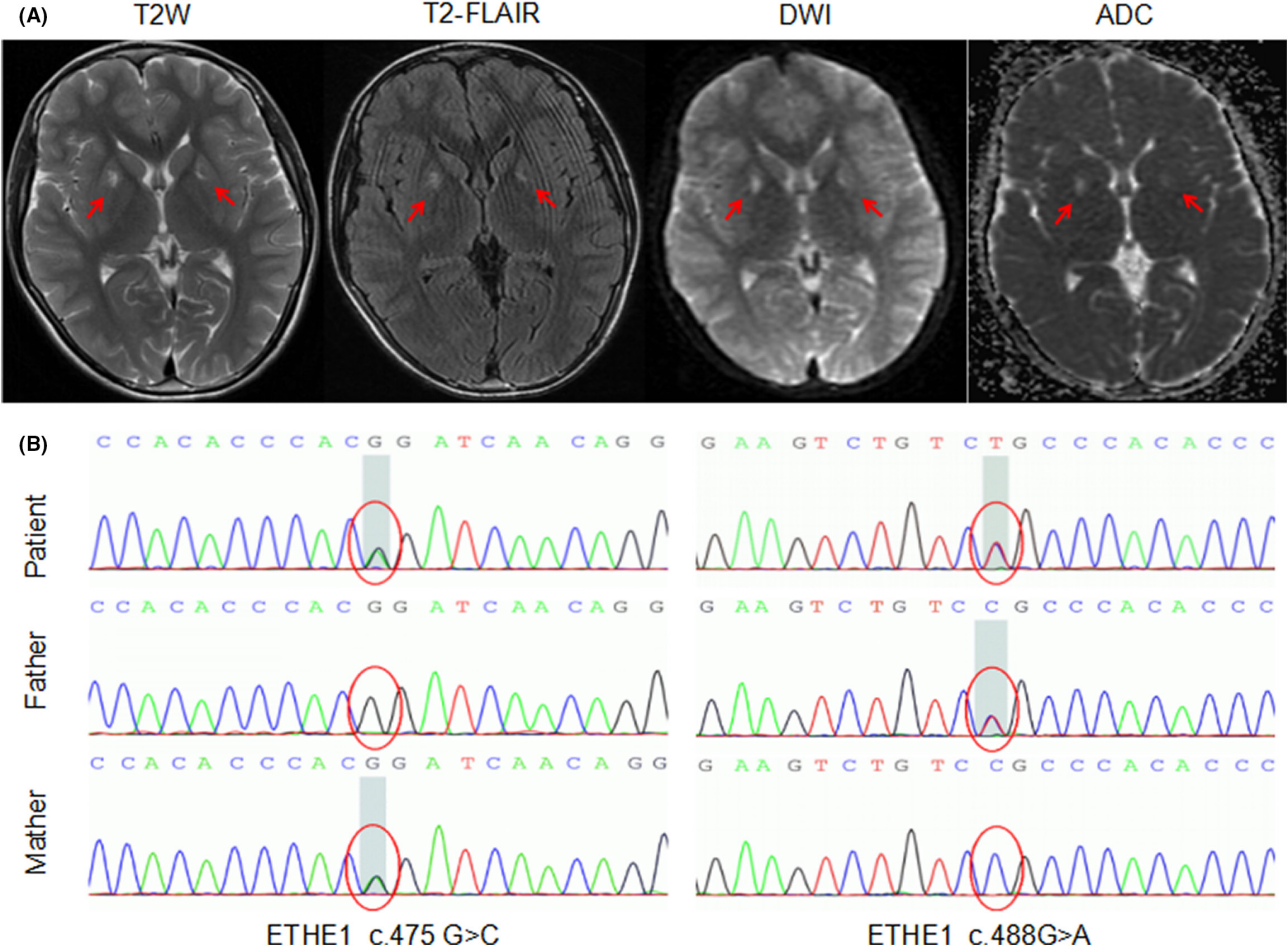
MRI and genetic observations in a patient with ethylmalonic encephalopathy. (A) T2‐weighted, T2 fluid‐attenuated inversion recovery, diffusion‐weighted MR, and associated apparent diffusion coefficient MR of axial images of brain MRI show hyperintensities in the bilateral putamen (red arrows). (B) Sequence electropherogram of c.488G>A and c.475G>C (gray rectangles) variations in the patient from the father and mother, respectively.

**TABLE 1:** Nerve conduction study results

Nerve stimulated	Stimulation site	Recording site	Latency (ms)	Amplitude (motor, mV)	Distance (mm)	Conduction velocity (m/s)
R peroneal	Ankle		2.8	1.0		
	Ankle–fibular head	EDB	8.0	1.0	260	50
	Fibular head	TAM	1.7	17.9		
L peroneal	Ankle		3.6	1.9		
	Ankle–fibular head	EDB	8.6	1.8	250	50
	Fibular head	TAM	2.1	17.6		
R tibial	Ankle		2.6	31.7		
L tibial	Ankle		3.2	31.7		

Abbreviations: EDB, extensor digitorum brevis; TAB, tibialis anterior muscle.

The patient was considered to have a genetic disorder; therefore, genetic testing was performed on the patient and the parents. Exome sequencing revealed heterozygous mutations c.488G>A (p.R 163Q) and c.475G>C (p.R159C; novel) of ETHE1 in the patient (Figure 1B), which was validated by Sanger sequencing. An elevated EMA level, imaging presentation, and genetic diagnosis confirmed that the patient had EE. Because ETHE1 is a mitochondrial sulfur dioxygenase involved in the catabolism of sulfide that accumulates to toxic levels in ethylmalonic encephalopathy,^3^ we recommend that patients limit their dietary intake of sulfur‐containing amino acids, and an oral idebenone (90 mg/day) and mecobalamin (500 μg/day) treatment. The patient's lower limb weakness symptoms did not progress after 12 months. Because the patient did not have significant microvascular symptoms, we did not administer drugs that inhibit hydrogen sulfide production, such as metronidazole and N‐acetylcysteine, which have clear efficacy in other literature.^4^ Treatment is mostly supportive of EE; curative treatment has not been achieved, but gene therapy and liver transplantation have been encouraged for a more effective treatment of EE.^5^, ^6^ Patients with EE who have metabolic coma, continuous dialysis renal replacement therapy is a good option.^7^ At the present time, we are still following up on the long‐term prognosis of the patient.

Mutations in ETHE1 are the major cause of EE, with over 60 mutations reported and only approximately 100 patients diagnosed worldwide; most of these patients die in the first decade of life.^1^ EE can occur in majority of typical cases with a severe clinical presentation and a few cases with mild atypical findings such as spastic paraplegia, whose diagnosis is often delayed until adolescence or adulthood.^4^, ^7^ The same mutation (c.488G>A (p.R 163Q)) was described in other patients with EE who did not have mild symptoms similar to the present case.^8^ Moreover, although this is a single case report, a possible correlation between the novel c.475G>C (p.R159C) ETHE1 mutation and an atypical onset with slight peripheral nerve damage cannot be excluded.

To the best of our knowledge, this is the first case report that describes atypical clinical features with slight peripheral nerve damage in EE with a novel compound heterozygous c.475C>T (p.R159C) variant of ETHE1, expanding the neurological and genetic phenotypes of this rare disease. The pathogenesis of atypical EE with mild neurological symptoms remains unknown. Whether a different compound heterozygous variant of ETHE1 in EE causes a different neuropathy remains to be explored in future experiments.

## CONFLICT OF INTEREST

The authors declare no conflict of interest.

## INFORMED CONSENT

We obtained patient permission and informed consent for publishing their information and images.

## Supporting information


Video S1

## Data Availability

Data available on request from the authors.
